# Eye Tracking for Assessment of Situational Awareness in Bridge Resource Management Training

**DOI:** 10.16910/jemr.12.3.7

**Published:** 2019-04-16

**Authors:** Oguz Atik

**Affiliations:** Dokuz Eylul University Maritime Faculty, Ïzmir, Turkey

**Keywords:** Eye movement, eye tracking, area of interest, individual differences, bridge resource management, situational awareness, maritime education, simulation and training

## Abstract

The purpose of this study is to experiment eye tracking in situational awareness assessment in Bridge Resource Management training of ship officers who play a critical role in maritime accidents. The maritime industry focuses on human factor developing and improving regulations including training requirements to prevent marine casualties. The mandatory Bridge Resource Management training as per international regulations includes assessment of situational awareness of trainees in full mission ship bridge simulators. The study involves capturing and analyzing eye movement data from maritime cadets and ship officers with sea experience in a simulation exercise. An eye tracking analysis software and eye tracking glasses are used for the study. Inferential and descriptive analyses were both used to validate the results. Significant differences were found between fixation duration measurements of novice cadets and experienced officers. Heat map visualizations also revealed differences in focuses of attention among participants. The evaluations of the certified simulator assessors are considered as the ground truth, and the results were compared to and discussed accordingly. The results show that the eye tracking technology is a valuable complementary tool for assessment of situational awareness in a simulator environment, utilized with the existing conventional observation and performance measurement methods. The study reveals that eye tracking provides the assessor with novel data in simulator based maritime training, such as focus of attention, which contributes to the evaluation of the situational awareness. The study, therefore, contributes to maritime education aiming to improve the effectiveness of Bridge Resource Management training. It also contributes to scientific research on eye movement in maritime field by proposing the integration of eye tracking in the Bridge Resource Management training.

## Introduction

The sea transport is one of the most important means of transportation and according to The United Nations Conference on Trade and Development (UNCTAD) [1], about 80% of the world trade volume, and more than 70% in value is carried out by shipping with an expected average annual growth of 3,2% by 2022. The world maritime traffic is growing, contributing to increasing challenges on safety at sea [2]. The research proves that the main contributing factor to marine casualties is human error, which accounts for 75 to 96% of various types of accidents: 84 to 88% of tanker accidents, 79% of towing vessels groundings, 89 to 96% of collisions, and 75% of fires and explosions [3]. Variations in situational awareness (SA) levels of the seafarers during critical tasks is frequently linked to human error [4]. Marine accident investigations have indicated that the accidents are frequently originated from the ships’ bridge operations, therefore increasing the SA of the bridge team is crucial [5]. Training and competency assessment of maritime officers in realistic simulators in improving SA is critical for maritime safety. The International Convention on Standards of Training, Certification and Watchkeeping for Seafarers (STCW) published by IMO [6], which adopts competency-based training principles [7], includes regulation guidelines on simulator-based maritime training, such as Bridge Resource Management (BRM) course. The 2010 Manila Amendments to STCW Convention and Code requires that competencies in both technical and non-technical skills, such as SA, be demonstrated by ship officers.

The purpose of this study is to develop an innovative approach to assess and improve the SA of the ship bridge team, and potentially contribute to maritime industry and safety. The study focuses on the assessment of SA of ship officers in BRM training. The study proposes the use of eye tacking technology as an assessment tool to enhance effectiveness of the training. The conventional assessment methods used in simulation training are limited to monitoring the participants from inside an instructor station intentionally separated from the simulation room where the participants complete the given tasks. The rooms are separated to create a realistic environment. At most, cameras and microphones are used to improve monitoring capabilities and live observation of mouse tracking provides additional data. However, the process is very limited in comparison to the data eye tracking provides. The conventional methods of observing and monitoring do not provide the assessor with any data on focus of attention, which enriches evaluation of the SA. Integrating the eye tracking method proposed in this study into BRM training will enable to determine the adequacy of SA of a BRM course trainee by the “simulator trainer and assessor” [6, 8]. This study aims to prove the importance and novelty of eye tracking by testing the system on maritime cadets and experienced ship officers in BRM training simulation exercise in a full mission ship bridge simulator.

## Bridge Resource Management Training

Bridge Resource Management (BRM), which has been used in the maritime industry for over a decade, is the maritime equivalent of Crew Resource Management (CRM) [9]. CRM, developed as a training concept by the aviation industry, is a management system involving use of all available means such as people, equipment, and process in order to improve safety following realization of the vital role of human factor and accidents. The purpose of CRM, which had been adopted for a variety of other high-reliability industries such as the nuclear, medical, and maritime industry, was to improve crews’ non-technical skills in critical areas such as teamwork, leadership, decision-making, and SA [10, 11]. The non-technical skills are the cognitive, social and personal resource skills that complement technical skills and are vital for safety and efficiency [12].

The maritime industry has adopted aviation’s CRM training as the accident research focused on the human involvement in maritime accidents [13]. Hetherington et al. [9] point out, in their research on human element in maritime safety, factors such as fatigue, stress, health, teamwork, decision-making, communication, and SA as contributory causes of accidents. Sandhåland et al. [14], in their study examining the collisions between attendant vessels and offshore facilities on the Norwegian continental shelf, reveal that 18 of the 23 cases of collisions preceded by loss of SA on the bridge. They claim that “the bridge crew must be able to identify key aspects of the environment accurately, understanding the meaning of what they sense, and have a good sense of what can happen”. BRM training, which is a simulator-based practical training, aims to improve maritime safety by reducing human error.

Cross & Muirhead [15] define the BRM training outcomes as; full use of internal and external resources of a vessel, clarifying seafarers’ respective responsibilities and obligations in bridge operations, effective use of soft skills and hard skills in different situations, effective use of facilities of the bridge to maintain safe navigation, reducing potential human errors, and implementing emergency procedures in different situations. The International Maritime Organization (IMO) requires all ship officers to demonstrate knowledge of BRM principles to be certified or to renew their certificates and according to the STCW Code under the section for “Master and deck department” SA is one of the areas to be addressed in the context of BRM [6]. BRM training enables to evaluate and improve officer’s awareness of the ship’s systems and of external factors and skills in collecting relevant information and identifying hazards [16].

## Assessment of Situational Awareness

Situational awareness (SA) is the perception of the elements in the environment, the comprehension of their meaning, and the projection of their status in the near future [17]. SA is the accurate perception of the elements at sea such as; dangers, and other ships as well as the comprehension and projection of their status in the near future [18]. SA is very critical for the bridge team [19]. Zhang [18] indicates that SA is very important for any professional competence and defines it as the most critical element of BRM, forming the basis for decision making and performance of hard skills. Cordon, Mestre, & Walliser [20], cited by Wahl & Kongsvik [16], describe SA as “a key factor in navigation influencing officers’ attention, spatial aptitude, organizing, decisions and awareness”. According to Wahl & Kongsvik [16], SA is ability to have a situational overview and to fit this knowledge into a mental model for problem recognition.

The main categories of SA assessment methods include freeze-probe recall techniques, real-time probe techniques, post-trial self-rating techniques, observer rating techniques, performance measures, and process indices [21, 22]. Among these methods, observer rating technique and performance measures are recommended and mainly used by the “simulator trainer and assessor” in Bridge Resource Management training in maritime education [6, 8]. The main advantage of these methods is their non-intrusive nature however, observer rating technique is vulnerable to subjective judgments, and the issue with the performance measurement technique is that an experienced trainee may perform well even when his/her SA is inadequate. In contrast, due to lack of experience, a novice trainee with a higher level of SA may fail a task. Asyali [23] indicates assessment of competencies in non- technical and technical skills during simulator training should optimize objective process, and ensure that subjective judgments are minimized. Two similar studies on the visual attention of anesthetists, cited by Williams, Quested, & Cooper [24] shows great discrepancy between assessment methods. Ford et al. [25] covertly videotaped and reported that subjects spend 5% of their time looking at the patient monitor. Schulz et al. [26] used eye tracking for the assessment of individual's distribution of visual attention as an underlying process for attaining adequate SA and suggested that the subjects spent 30% of their time looking at the patient monitor. Williams, Quested, & Cooper [24] indicate that it is inherently difficult to observe visual attention and “observation techniques require the time and expense of expert observers, who may bring with them their own experiences, ideas, attitudes, and biases”. Considering that observation is the principal assessment technique in BRM training [6, 8], eye tracking is a valuable tool in maritime education, allowing to assess objectively and continuously without interrupting the activity [27]. Eye tracking during task execution is an example of process indices methods; useful in determining SA in field settings created in simulators and can be used to evaluate how the trainee’s attention is allocated [22, 28, 29], as cited by Babu et al. [30], concluded in their eye movement study on an unmanned aerial vehicle that “visual attention allocation” and “visual scanning” are major indicators of situational awareness.

## Eye Tracking Technology

Recent technological developments in biomedical field come into use in the human factor and ergonomics research arena has led to expanding use of various tools such as Electroencephalography (EEG), functional NearInfrared imaging (fNIR), Galvanic Skin Response (GSR) and eye tracking which could be used to understand the cognitive behavior of human in variety of industries. Eye tracking is the method used to measure eye movements and point of gaze with special equipment commonly called an “eye tracker”. There are four common methods used to measure eye movements, including the use or measurement of Electro-OculoGraphy (EOG), Scleral contact lens/search coil, Photo-OculoGraphy (POG) or Video-OculoGraphy (VOG), and Video-based combined pupil and corneal reflection [31, 32, 33].

While the Electro-OculoGraphy (EOG) method relies on measurement of the skin’s electric potential differences, Scleral contact lens/search coil relies on an optical or mechanical reference object mounted on a contact lens. Photo-OculoGraphy (POG) or Video-OculoGraphy (VOG) includes many eye movement recording techniques for measurement of distinguishable features of the eyes [33, 34, 35].

Video-based combined pupil and corneal reflection tracker, which is the most basic form of eye tracking methods, determines the focal points in the visual field with fixations and saccades using cameras and other hardware [31, 33]. Some measurements such as pupil diameter, frequency of blinks, duration of blinks, and number of blinks are also used for deeper analysis of cognitive processing and stress in this system [36].

The usability of the eye tracker is appraised by metrics that are relevant to the duties and their inherent cognitive activities. For example, distribution of fixation has been used as a measurement of mental workload by Di Nocera et al. [37, 38], whereas fixation time has been used to determine the importance of giving the eye time to actually look for objects in the surroundings by Hareide & Ostnes [39].

Researchers focus on eye tracking technology in variety of disciplines, such as the medical field [40, 41], marketing [42], usability research [43], information technologies [44], agriculture [45], multimedia technology [46, 47], education [48, 49] and aviation [30, 50, 51]. Review of human factor research in marine transportation field shows that a limited number of studies have been carried out on eye tracking of ship officers, especially on education and training, considering other related fields such as; the aviation industry. Kum, Furusho, & Arslan [52] have collected fixation data from maritime cadets on bridge simulator using an “eye mark recorder” and obtained findings on relationship between experience and focus of attention. Lutzhoft & Dukie [53] have studied the focus of attention and fixation of ship officers during watchkeeping, aiming to contribute to safe navigation. In the study conducted by Forsman et al. [54], the behavior of novice and expert boat drivers have been tested during high speed navigation at sea. Gaze behavior from both novices and experts has been investigated with respect to direction, object and distance of fixations. Muczynski & Gucma [55] have used eye tracking for their research on the human factor in marine operations. Hareide & Ostnes [56] have tested the use of eye tracking technology in marine transportation focusing on Integrated Bridge System and human-machine interaction. Di Nocera et al. [38] have studied fatigue and attention using eye trackers in simulators. Atik & Arslan [57] have revealed that the eye tracking technology can be a valuable tool for assessment of electronic navigation competency as a part of maritime training. Thus, this study presents a novelty emphasizing the potential contribution of eye tracking technology in BRM training evaluation required by STCW.

It is important that the simulator can reflect real events. The ability to control, record and play the scene for evaluation and debriefing are important features of the simulators. Observing, monitoring, and recording the activities of the trainee are essential steps in simulator training highlighted in the STCW Code section A-I / 12 [6]. Eye tracking, as a valuable research tool for observing interaction of people with visual information [58], is used in this study in observing, monitoring, and recording activities in simulator training as an assessment tool. Eye tracking provides the assessor with the ability of recording the trainee’s gaze, fixation, and attention as well as live observation of the trainee’s activities during a simulation scenario. The traditional observation methods, which are currently used in simulator-based maritime training, are limited in collecting objective behavioral data that, according to Ali-Hasan, Harrington, & Richman [59], as quoted by Choi et al. [47], can be obtained by capturing the eye movement patterns of a person using the eye tracking technique.

## Methods

**Figure 1:**
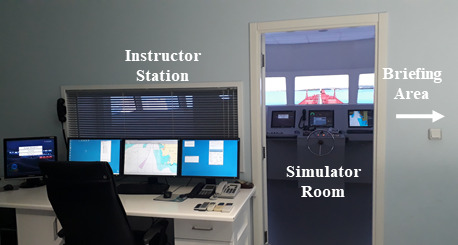
Full mission bridge simulator.

The experiment was conducted in the full mission bridge simulator of Dokuz Eylul University Maritime Faculty. The bridge simulator used consists of a ship station having a full scale mock-up of a ship's bridge with instruments for navigation, and a separate instructor and debriefing room equipped with equipment necessary to monitor the activities inside the bridge effectively as per STCW (Figure 1).

**Figure 2:**
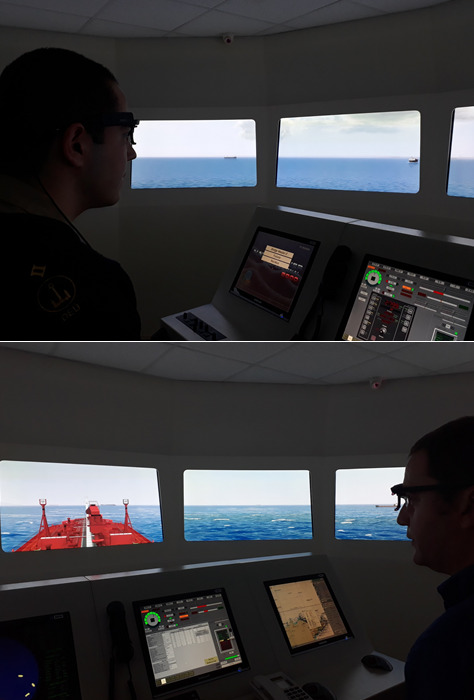
A participant with the eye tracking glasses executing a simulation exercise in the full mission bridge simulator.

The subject group consist of 15 freshman cadets, 15 junior cadets and 15 oceangoing deck officers with sea experience. The officer participants were considered as expert sample group while junior and freshman cadets were selected as novice. However, juniors have already been onboard ships twice on their freshman and sophomore years, while freshmen have had no sea experience. Both junior and freshman cadets have taken watchkeeping courses including “International Regulations for Preventing Collision at Sea” (Colregs, 1972) [60], which was required to execute the simulator exercise. A total of 45 eye tracking recordings in the full mission bridge simulator (Figure 2) were captured using Tobii Pro Glasses 2 with gaze sampling frequency of 100 Hz. The glasses were calibrated for each participant before recordings. The eye tracking data obtained from 35 participants, including 15 freshmen, 10 junior, and 10 officer, was analyzed using Tobii Pro Lab Analyzer software to obtain metric data. 10 recordings out of 45 were not suitable to be analyzed by the Tobii Pro Lab Analyzer software due to the dynamic nature of the simulation exercise and the participants’ activities inside the simulator room. The metric data from 35 participants were analyzed using Statistical Package for the Social Sciences (SPSS). All 45 recordings were examined in detail and evaluated by 2 certified simulator trainer and assessor for qualitative analysis purposes.

The simulation scenario was designed as a common BRM exercise to test the SA of the participants in a heavy traffic environment which required proper look-out competence and determining and avoiding risk of collision. The exercise scenario was planned to be completed in 13 minutes and all 45 participants’ eye movements were recorded throughout the exercise, while observed live during the exercise and coded for later in-depth evaluation by the certified simulator trainers. Evaluations of the simulator assessors, including debriefings, were considered as the ground truth / baseline and compared to the metric results and the heat map visualizations. In this study, the main focus was to test the eye tracking system’s usability as a supportive and complementary method to the observation method conventionally used in maritime simulation training. In contrary to the flight simulator training, which is compared to and followed by the maritime educators and researchers as many other aspects of the aviation industry, being the pioneer especially in safety related practices, in the ship bridge simulators the situations develop relatively slow. The characteristics of the maritime traffic and the low sailing speeds of the vessels require specific set of rules for creating scenarios and assessing the trainees. Variety of technical and non-technical skills are evaluated in a BRM exercise and visual observation of the traffic, which requires proper look-out, is one of the most critical skills expected from a trainee, and considered as one of the evaluation criteria for SA in a conventional assessment process. It is possible to assess the three levels of situational awareness which are perception, comprehension, and projection by observing the look-out skills and the actions taken by the trainee, with limitations, and eye tracking is proposed to fill in the gap. The scenario in this study was constructed accordingly.

**Figure 3:**
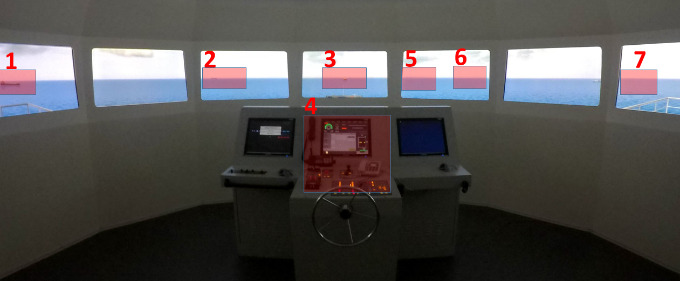
Areas of interest in the bridge simulator.

A BRM simulation scenario was created, involving determining risk of collision and decision making by the watchkeeping officer (participant), which required adequate SA. The scenario was designed so that there was actually no risk of collision existed which would require any action by the participant. However, though not necessary, a minor alteration of ship’s course to starboard is acceptable as per Colregs (1972) [60] to pass even at a safer distance from the target vessels. Although the scenario involved no risk of collision, it was designed as a high density traffic situation for any watch officer, and constant look-out and evaluation of the situation (perception, comprehension, and projection) was necessary, which is considered high work load environment at sea. The areas of interest (AOI) are the target vessels; one on the port beam, a give-way vessel on the port quarter, which would alter course and pass clear of the own ship; a ship dead ahead at a safe distance at same speed on same course with the own ship; several anchored vessels at a close distance on the starboard quarter; a vessel on the starboard quarter which was not on a collision course, but would be a stand-on vessel if considered risk of collision; and a vessel on the starboard beam. The conning display with the own ship’s navigational data such as; course, speed, rudder angle, engine status, and autopilot controls, which the participants were free to adjust, was designated as another AOI to evaluate the participants’ visual attention on ship’s controls (Figure 3, Table 1). The participants were instructed not to operate the radar, which was turned off by the trainer, and navigate safely towards the port visible on the bow considering themselves officer on watch onboard a ship.

**Table 1:** List of areas of interest.

AOI		Description
1	Port Beam	A target ship on port beam on a safe course to pass on the stern
2	Give way Vessel	A give-way vessel on port quarter which alters course to pass safely on the stern
3	Ahead	A vessel dead ahead
4	Conning	Conning display, radar, and ecdis
5	Anchored Vessels	Anchored vessels on starboard quarter
6	Stand-on Vessel	A vessel on starboard quarter on a safe course to pass on the bow
7	Starboard Beam	A target ship on starboard beam on a safe course to pass on the stern

The numbers on the AOI figure (Figure 3) indicate AOI and corresponding descriptions on Table 1.

Three methods were used in the study and compared to the ground truth. Visual inspection of the eye gaze recordings by the assessors included all 45 participants for the full duration of each exercise. Heat map visualizations were used to analyze focus of attention. Fixation duration measurements were used for statistical analysis. Eye movements until the first action taken by the participants in the first 5 minutes of the exercise were used for the analysis of the metric data. After the first 5 minutes, the areas of interest overlapped due to the dynamic nature of the exercise, which made it impracticable for software analysis. The exercise was designed to experiment eye tracking both in static and dynamic environment. Although the participants’ physical activity does not have any negative effects on capturing the eye movements and instantaneous observation of focus of attention of the participants by the assessor while the exercise runs, it appears to affect the analysis process of the Tobii software. The high level of head and body motion inside the simulator room, which has 225 degrees visual layout, caused deficiencies in the analysis of the eye movement data and were detected during the qualitative comparison with the recordings. The recording of 5 officers and 5 juniors were not properly analyzed by the software and were not used in the statistical analysis and the heat map visualization presentations. The qualitative comparison of the eye movement recordings and the analyzed data also revealed issues on the AOI port beam and the starboard beam. The potential software image processing issue was overcome by analyzing the concerned AOI separately.

Total fixation duration, which is the time the eye remains focusing on an AOI [61], were analyzed for the purpose of this study. Total fixation duration shows the total amount of time the participant is fixated on a specific AOI during the simulation scenario. Fixation duration was used because it can indicate cognitive processing on an AOI [61, 62]. Descriptive and inferential analyses were both used to evaluate the eye tracking data collected. Evaluation of recordings by certified simulator trainer and assessors were used to complement the results. The non-parametric Kruskal-Wallis and Mann-Whitney U tests were used to test the research hypotheses, which predicted that there would be significant differences between the experienced officer participants’, junior cadets’, and freshman cadets’ fixation duration measurements on the specified areas of interest. The means of the participants’ fixation duration data were compared for the purposes of descriptive analysis.

This research complied with the American Psychological Association Code of Ethics and informed consent was obtained from each participant.

## Results

The Shapiro-Wilk test of normality was conducted for the statistical analysis [63, 64] and the results showed that the conditions for a normally distributed data (p > .05) were not met. A non-parametric Kruskal-Wallis test, on 95% confidence level, was conducted to examine the differences on fixation durations of officer (n=10), junior cadet (n=10) and freshman cadet (n=15) participants and significant differences were found among participants’ fixation durations on the areas of interest port beam, give way vessel, ahead, anchored vessels and stand-on vessel (Table 2).

**Table 2:** Kruskal Wallis Test Results for total fixation durations of officer, junior, and freshman participants.

AOI	Test Results	Mean Rank	
Port Beam	H(2)=6.47 p=0.04	Officer Junior Freshman	24.90 15.75 14.90
Give way Vessel	H(2)=17.45 p=0.00	Officer Junior Freshman	25.65 22.70 9.77
Ahead	H(2)=8.31 p=0.02	Officer Junior Freshman	23.00 21.60 12.27
Conning	H(2)=3.89 p=0.14	Officer Junior Freshman	15.85 23.40 15.83
Anchored Vessels	H(2)=14.06 p=0.00	Officer Junior Freshman	27.10 18.60 11.53
Stand-on Vessel	H(2)=17.78 p=0.00	Officer Junior Freshman	27.70 19.55 10.50
Starboard Beam	H(2)=2.61 p=0.27	Officer Junior Freshman	19.50 21.20 14.87

A non-parametric two-tailed Mann-Whitney U test on 95% confidence level, which does not require normally distributed data [65], was run to determine the differences between fixation durations of officers and juniors, officers and freshmen, juniors and freshmen on the designated areas of interest to contemplate the Kruskal Wallis test results.

The Mann-Whitney U test run indicated that there are significant differences between officer and junior participants’ fixation duration measurements on AOI port beam, anchored vessels, and stand-on vessel (Table 3). There is no significant difference between measurements on AOI conning due to the critical value of U (24) found to be 23 for officers (n=10) and juniors (n=10) for 95% confidence level [66].

**Table 3:** Mann-Whitney U Test Results for total fixation durations of officer and junior participants.

AOI	Participant	Test Results
Port Beam	Officer (Mdn = 5.01) Junior (Mdn = 1.08)	U = 17.00, p = .013, r = 0.56
Give way Vessel	Officer (Mdn = 9.49) Junior (Mdn = 5.10)	U = 36.00, p = .29, r = 0.24
Ahead	Officer (Mdn = 47.53) Junior (Mdn = 32.23)	U = 44.00, p = .65, r = 0.11
Conning	Officer (Mdn = 6,85) Junior (Mdn = 30.72)	U = 24.00, p = .049, r = 0.44
Anchored Vessels	Officer (Mdn = 19.02) Junior (Mdn = 3.97)	U = 19.00, p = .019, r = 0.53
Stand-on Vessel	Officer (Mdn = 15.21) Junior (Mdn = 1.62)	U = 20.00, p = .023, r = 0.51
Starboard Beam	Officer (Mdn = 1.23) Junior (Mdn = 2.20)	U = 45.00, p = .705, r = 0.09

The Mann-Whitney U test showed that there are significant differences between officer and freshman participants’ fixation duration measurements on AOI port beam, give-way vessel, ahead, anchored vessel, and stand-on vessel (Table 4).

**Table 4:** Mann-Whitney U Test Results for total fixation durations of officer and freshman participants.

AOI	Participant	Test Results
Port Beam	Officer (Mdn = 5.01) Freshman (Mdn=0.34)	U = 39.00, p = .044, r = 0.41
Give way Vessel	Officer (Mdn = 9.49) Freshman (Mdn=0.56)	U = 12.50, p = .00, r = 0.70
Ahead	Officer (Mdn = 47.53) Freshman (Mdn=4.28)	U = 31.00, p = .015, r = 0.49
Conning	Officer (Mdn = 6.85) Freshman (Mdn=6.62)	U = 70.50, p = .80, r = 0.05
Anchored Vessels	Officer (Mdn = 19.02) Freshman (Mdn=0.00)	U = 15.00, p = .001, r = 0.68
Stand-on Vessel	Officer (Mdn = 15.21) Freshman (Mdn=0.00)	U = 8.00, p = .000, r = 0.68
Starboard Beam	Officer (Mdn = 1.23) Freshman (Mdn=1.24)	U = 55.00, p = .262, r = 0.23

The Mann-Whitney U test indicated that there are significant differences between junior and freshman participants’ fixation duration measurements on AOI give-way vessel, ahead, anchored vessels, and stand-on vessel (Table 5).

**Table 5:** Mann-Whitney U Test Results for total fixation durations of junior and freshman participants.

AOI	Participant	Test Results
Port Beam	Junior (Mdn = 1.08) Freshman (Mdn=0.34)	U = 64.50, p = .554, r = 0.12
Give way Vessel	Junior (Mdn = 5.10) Freshman (Mdn=0.56)	U = 14.00, p = .001, r = 0.69
Ahead	Junior (Mdn = 32.23) Freshman (Mdn=4.28)	U = 33.00, p = .020, r = 0.47
Conning	Junior (Mdn = 30.72) Freshman (Mdn=6.62)	U = 47.00, p = .120, r = 0.31
Anchored Vessels	Junior (Mdn = 3.97) Freshman (Mdn=0.00)	U = 38.00, p = .037, r = 0.42
Stand-on Vessel	Junior (Mdn = 1.62) Freshman (Mdn=0.00)	U = 29.50, p = .008, r = 0.53
Starboard Beam	Junior (Mdn = 2.20) Freshman (Mdn=1.24)	U = 48.00, p = .130, r = 0.31

**Figure 4:**
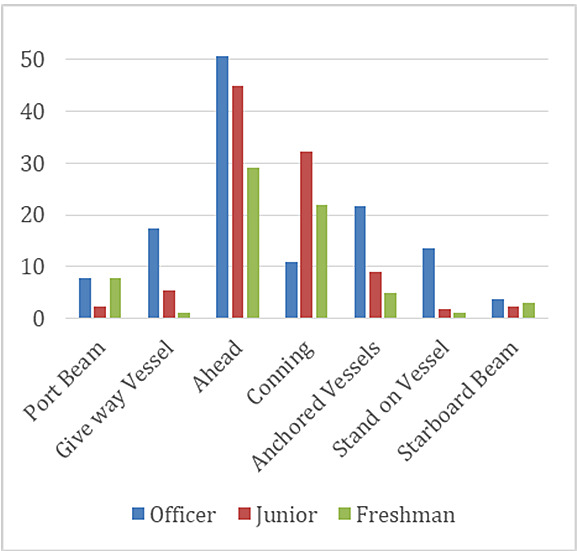
Comparison of means of total fixation duration measurements of officer, junior, and freshman participants.

The means of fixation duration measurements on all areas of interest except conning are larger for the officer participants than for the junior and freshman participants. The officers fixated on conning less than both juniors and freshmen. The measurements of the junior participants were larger than the freshmen on the AOI; give-way vessel, conning, ahead, anchored vessels, and stand on vessel. The measurements of the freshman participants were larger than the juniors on the AOI; port beam and starboard beam (Figure 4).

**Figure 5:**
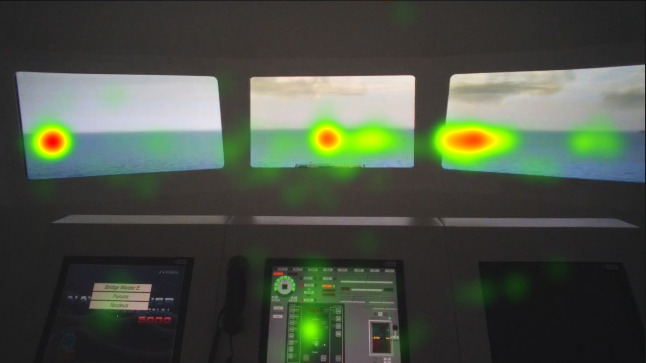
Heat map visualization of officers on AOI 2-6.

**Figure 6:**
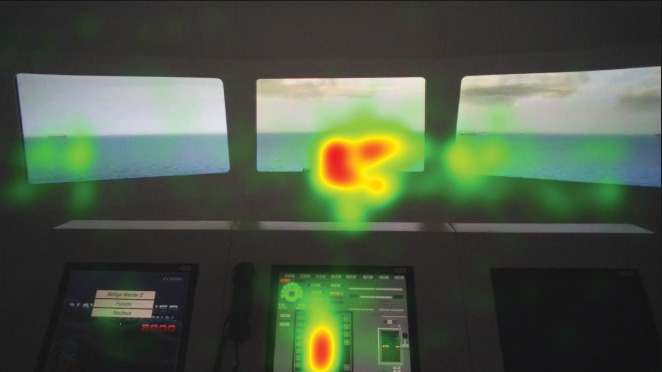
Heat map visualization of juniors on AOI 2-6.

**Figure 7:**
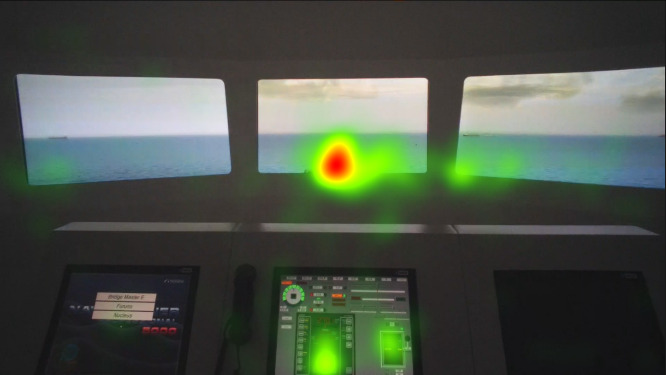
Heat map visualization of freshmen on AOI 2-6.

The heat map visualizations of the participants in Figures 5, 6, 7 are presented for comparison of focus of attention between groups. Heat maps indicate that the officer’s attention is mainly focused on AOI give way vessel, ahead, and the anchored vessels. They also monitored the stand-on vessel approaching from the starboard side. Fixation on the conning is also visible on the heat map. Juniors mainly fixated on the AOI ahead and they spend significant amount with the conning, appears to be specifically the autopilot. Freshmen were focused on mainly AOI ahead and overlooked the other targets. Their fixation on the conning, especially on the autopilot is shown on the heat map.

**Figure 8:**
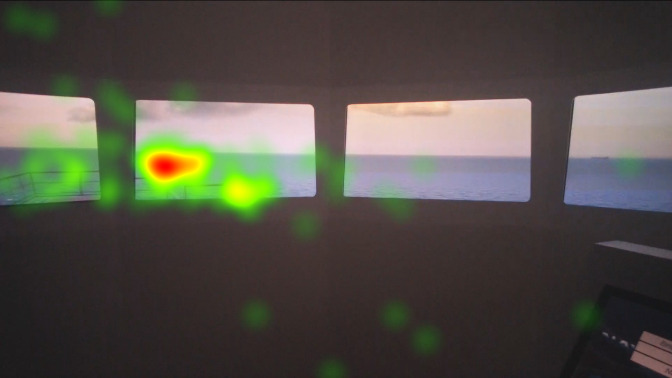
Heat map visualization of officers on AOI 1.

**Figure 9:**
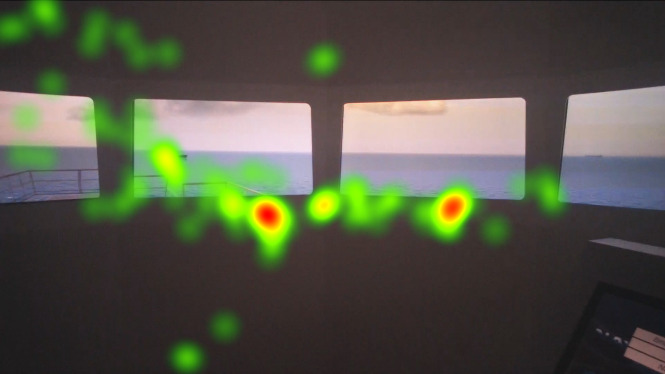
Heat map visualization of juniors on AOI 1.

Heat maps of AOI port beam, which as mentioned in the methods section was analyzed separately similar to the AOI starboard beam, in Figure 8, 9, and 10 indicate that the juniors’ and freshmen’s fixations are relatively scattered, while the officers’ mainly cumulated on the target. The port and starboard beam heat maps should not be compared to the heat maps of the other AOI or each other, since they were analyzed separately.

**Figure 10:**
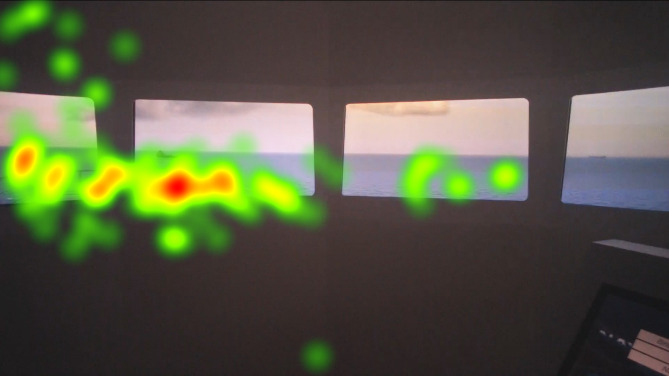
Heat map visualization of freshmen on AOI 1.

**Figure 11:**
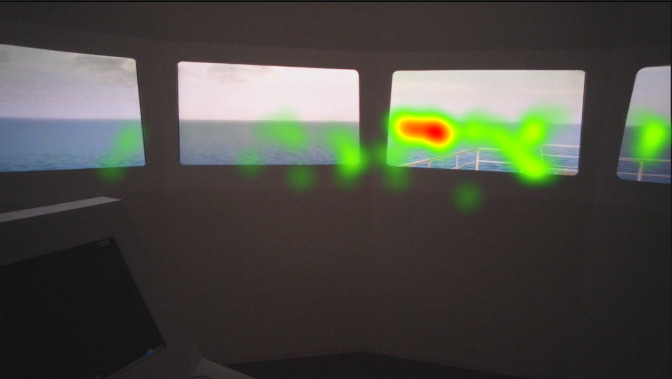
Heat map visualization of officers on AOI 7.

**Figure 12:**
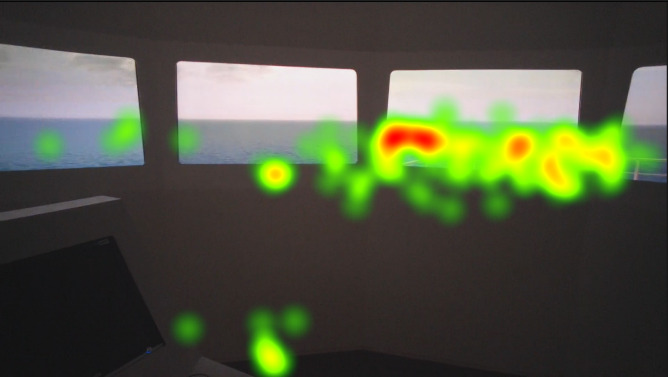
Heat map visualization of juniors on AOI 7.

In the heat maps of AOI starboard in Figure 11, 12, and 13 indicate that the officers’ and freshmen’s fixations are similarly, mainly cumulated on the target, while the juniors’ scattered.

**Figure 13:**
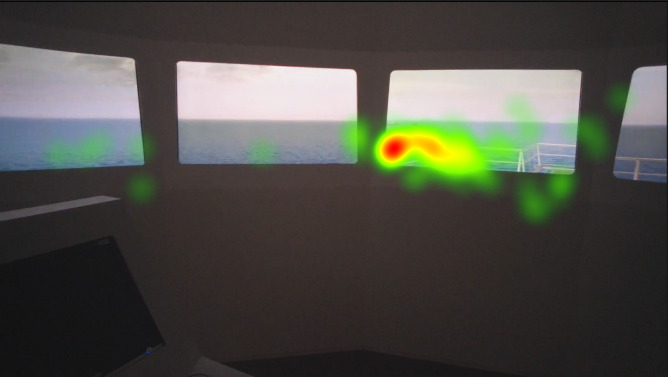
Heat map visualization of freshmen on AOI 7.

**Ground Truth:** Currently, in a BRM course, assessment of the SA conducted merely through visual and audial observations of the certified “simulator trainer and assessor” is the accepted method according to STCW. Debriefing at the end of an exercise is a complementary component of the assessing process. In this study, assessment of the certified assessors is considered the baseline / ground truth and the results were compared to and discussed accordingly. In a full mission bridge simulator, several tools are used for assessment such as; cameras to visually observe the trainees and their actions and follow the proceedings of an exercise, microphones to monitor the bridge communication, and repeater monitors to observe and evaluate the use of the electronic navigation equipment by the trainees. In the exercise run in this study there was no audible data collected and the electronic navigation equipment were off. The assessments were conducted by two experienced certified simulator trainer and assessor via visually monitoring the participants through the simulator room cameras and monitor the actions taken. In a conventional BRM assessment through observation and performance evaluation, decision making and actions are some of the key elements to evaluate SA. Participants who both carried out proper look-out, evaluation of which was based on camera monitoring, and avoided unnecessary actions were considered to have the full appraisal of the situation and having adequate SA. Table 6 summarizes the evaluations by the assessors including the observations, performance assessments, and debriefing input. The table also presents the quality of eye tracking recordings, and the software analysis for obtaining the metrics, which appears to correlate with the physical activities of the participants, such as walking and pacing up and down inside the simulator room, a common behavior of officers on watch at sea. The discussions of the study include the participants’ assessments, based on the ground truth, of which the software analysis was successfully conducted.

**Table 6:** Summary of the exercise evaluation based on observation and performance monitoring

		Officer	Junior	Freshman
**Course change**	**Port**	**1**	4	2
	**Stbd**	**1**	1	7
**Proper look-out**		14	11	6
**Adequate SA**		13	9	6
**High physical activity**		5	5	0
**Sound eye movement recording**		15	15	15
**Software analysis**		10	10	15

## Discussion

This study aims to prove the value and practicability of eye tracking in BRM training as a complementary assessment tool to be used along with the existing methods, and proposes the integration of the tool into BRM courses. The conventional monitoring and observation methods used in simulator-based maritime training are limited in assessing the participants’ SA, in that sense eye tracking fills in the gap. This novice-expert comparison study is designed to prove that the proposed assessment method, where the trainees can be evaluated with an additional input of eye trackers to the existing assessment methods, can be used in BRM training, where the trainees (novice) are expected to perform at an expert level after having completed the training. Monitoring the activities inside the simulator room from an instructor station has limitations which can be overcome utilizing the eye trackers. Use of eye trackers with the existing monitoring tools provides the assessor with the data of focus of attention of the trainee which brings a great value and depth to the assessment of SA for the assessor. The instantaneous eye tracking in addition to the existing monitoring tools during an exercise, rather than collecting data for future analysis, is the key advantage of the eye tracking for a maritime simulator assessor which is the main focus of this study. An interpretation of visual attention input provided by eye tracking for a specific target is a major contribution to SA assessment in a ship bridge simulator. In this sense, the potential of eye tracking in BRM training is extensive. Considering the long durations of a maritime simulation exercise due to its slow and gradually developing nature, observation is critical for the assessor and eye tracking enables the assessor to observe the trainee from a different perspective and evaluate his/her SA.

The evaluation based on observation and performance monitoring, inspection of the eye movement recordings, heat map visualizations, debriefing records, and the statistical results, which validate the research hypothesis, which have predicted that there would be significant differences between officer, junior, and freshman participants’ fixation duration measurements, were found to be compatible, and are discussed in this section.

The study revealed that the officers mostly maintained a proper look-out as per Colregs (1972) [60] as expected, and mainly kept their courses steady resulting in longer fixation duration measurements. According to Colregs (1972) [60] “Every vessel shall at all times maintain a proper look-out by sight and hearing as well as by all available means appropriate in the prevailing circumstances and conditions so as to make a full appraisal of the situation and/or the risk of collision”. The freshman participants took early action, some unsafe, without adequately focusing on the target vessels to be able to comprehend the situation. Visual tracking of targets by proper look-out to comprehend and project the situation is the most important duty of an officer on watch, and especially freshman underperformed in making “full appraisal of the situation”; or in other words in maintaining adequate SA.

The target vessels on port and starboard beam were relatively easier to determine that they do not involve any risk of collision. However, as per Colregs (1972) [60], all targets should be carefully checked for any change in the situation until finally having past and cleared. The fixation duration measurements of officers and freshmen on the port and starboard targets were close, while juniors’ attention was much less on the target on the port beam, supported by the heat maps. Heat maps also shows that officers were much more focused on the target and the freshmen’s fixations were scattered. The freshmen, confirmed by the debriefings, were struggling to comprehend and project the situation, while juniors show a sign of complacency, a common issue observed among junior and senior cadets.

The give-way vessel was set on a course approaching from port quarter to pass on own ship’s stern as per Colregs (1972) [60]. She required continuous attention until the participant confirmed her safe passage. The officer participants’ fixation duration differed from that of the freshmen as the freshmen did not properly observed the vessel and some altered the course to starboard while some to port, which is a wrong action to take. The alteration of course to port is never an option in such a situation, as per Colregs Rule 17/c [60], unless it is “special circumstance” such as an emergency, even the give-way vessel does not take a proper action. Supported by the debriefings, unnecessary action taken by the freshmen revealed lack of SA. When the situation was discussed in the debriefings, the freshmen who altered course to port stated that they were in doubt of the status of the “give way” vessel and tried to avoid collision by altering course to port since the starboard side was even more congested. The focus of attention of the officers on the give way vessel is clearly distinguished on the heat maps. The inferential results did not indicate a significant difference between measurements of the officers and the juniors on AOI give-way vessel, there is still a considerable difference between them according to the comparison of the means.

There is a significant difference between the measurements of the officers and the freshmen on AOI ahead. However, the heat maps show all groups kept adequate look-out dead ahead. The vessel dead ahead was set to sail at the same speed on the same course as the own ship, without any risk of collision, however she required continuous attention since positioned dead ahead. It requires constant observation to determine a target’s status dead ahead on the same speed and course, so if her speed or course changes, the officer on watch can react immediately to the changes in the situation. Proper look-out without unnecessary action is considered as adequate SA as explained in ground truth section.

Area of interest conning is the only AOI the officer participants paid less visual attention to. The results, confirmed by the debriefings, reveal that the experienced officers are well aware of the necessity for a proper look-out in a dense traffic situation. The officers’ visual attention was focused on the targets, and as they achieve adequate SA they did not feel the need to check the conning displays as much. The juniors focused on the conning display, specifically on the autopilot, according to the heat maps, which is an important data that could not be obtained without eye tracking. Debriefings show that juniors, having taken an electronic navigation course and being at sea before, felt need to check the equipment to control the situation. Freshmen were not familiar with the equipment and had less attention on the conning than juniors. The baseline here is, the conning had no function in this particular scenario, and needed no significant attention to assess the situation. The unnecessary fixation on the conning was accepted as indication of inadequate SA. While there is no significant difference between measurements of the participants statistically, eye tracking data provides valuable data, supported by the conventional methods.

The anchored vessels on the starboard quarter at a close range did not involve risk of collision but required continuous attention for any change in status. The tests indicate significant differences between all participants’ measurements on AOI anchored vessels. The officers properly kept their attention on the anchored vessels, clearly presented in the heat maps, while the juniors and the freshmen appear to have less attention on the same targets. The cadets took early unnecessary actions mentioned in the ground truth section, without achieving adequate SA by proper look-out. The debriefings reveal that junior and freshmen had difficulty in comprehending and projecting the situation. Without the radar input, achieving adequate SA, merely by visual data, requires experience.

The stand-on vessel was actually planned to be the most critical target for the participants, being the vessel to give way if considered to involve risk of collision, as per regulations. When a risk of collision exists, the stand-on vessel approaching from the starboard quarter towards the bow, as planned in the scenario, is required to keep her course and speed steady while the give-way vessel can maneuver to pass safely. While the officers had much larger fixation duration on the AOI than the juniors and the freshmen, as shown in the heat maps as well, the results indicate lower focus of attention on the stand-on vessel than the anchored vessels, which was not expected in the design of the scenario. The officers kept attention on the target to take immediate action, if required in case of a change in situation, however the debriefings reveal that the anchored vessels being much closer and congested, created more concern and considered to have higher risk of collision. The juniors and the freshmen considered the stand-on vessel being at a safe distance, and they were more concerned about taking immediate action for the vessel ahead, the anchored vessels, and the give way vessel which they considered critical.

The study highlights the importance of training and sea experience for the safety of navigation. According to the results, there are considerable differences between SA levels of the experienced officers and novice cadets in general, which is expected. The cadets take a major “watchkeeping” course in their senior year which requires the integration of all the skill and knowledge they developed during their education. Sea experience improve their technical and non-technical skills even more, as they sail out at sea as professional deck officers. SA, which forms the basis for decision making process, is a skill to improve continuously with experience.

## Conclusion

The results show that the eye tracking technology can be a valuable tool for assessment of SA in BRM training. While analysis of the eye tracking metric data was conducted as a scientific method for the purpose of the study, live tracking of the eye movement, therefore observing the instant visual attention indicating SA during the execution of a simulation exercise is the main advantage of integrating eye tracking into BRM training course. In BRM training courses, the evaluation of a trainee, using technical and non-technical assessment sheets, is finished by the completion of the course, and live tracking and assessment of the eye movement in a structured assessment form, with expected criteria specified, can be considered as a component of the whole evaluation process. This study, comparing novice and experts in a ship bridge simulation exercise proves that eye tracking provides the data of focus and attention of the participants, which enables to determine the adequacy of a trainee’s SA, in a way that no other assessment and evaluation method can provide. The conventional “observation” method used by the simulator instructor is very limited in assessment of certain tasks in exercises because of the physical constraints and eye tracking has the potential to fill in this gap.

The cost of the eye tracking systems could be considered as a limitation for the eye movement studies and practices in maritime education. However, full mission bridge simulators are large investments, and considering their potentials, eye trackers should be integrated into the system in maritime institutions. Limitations of eye tracking to obtain metric data is not an issue in live/instant eye movement observation, which is emphasized and proposed as a useful and applicable complementary method in assessment process in this study, however the limitations of the analysis software in dynamic scenarios should be considered when designing exercises aiming to collect metric data, since the maritime environment and ships’ motions are unstable. Another limitation of eye tracking in maritime simulator training is the size and the wide visual lay out of the simulator room, which brings out the issue of possible high physical activity of the trainees inside the room which affects software analysis process. The simulator trainers and assessors should also be aware of intrusiveness of the wearable eye gaze tracker, as a potential risk of trainees consciously altering the eye gaze behavior, and take caution to implement proper briefings and monitoring procedures.

The research shows that the integration of eye tracking in simulation based maritime training would be truly useful in improving and enhancing effectiveness. Eye tracking has great potential to contribute to maritime simulator training, and future studies should be conducted on other aspects of the training such as; ship handling, emergency situation handling, usability of navigational equipment, and ergonomics of bridge lay out.

## Ethics and Conflict of Interest

The author(s) declare(s) that the contents of the article are in agreement with the ethics described in http://biblio.unibe.ch/portale/elibrary/BOP/jemr/ethics.html and that there is no conflict of interest regarding the publication of this paper.
